# The collaborative research and service delivery partnership between the United States healthcare system and the U.S. Military Health System during the COVID-19 pandemic

**DOI:** 10.1186/s12961-022-00885-4

**Published:** 2022-07-19

**Authors:** Tracey Pérez Koehlmoos, Jessica Korona-Bailey, Miranda Lynn Janvrin, Cathaleen Madsen, Eric Schneider

**Affiliations:** 1 Uniformed Services University of the Health Sciences, 4301 Jones Bridge Road, Bethesda, MD 20814 United States of America; 2 The Henry M. Jackson Foundation for the Advancement of Military Medicine, Inc, 6720B Rockledge Drive, Suite 605, Bethesda, MD 20817 United States of America; 3 Yale University, New Haven, CT 06520 United States of America

**Keywords:** Military Health System, COVID-19 pandemic response, U.S. Healthcare System

## Abstract

**Objectives:**

To examine the military-civilian collaborative efforts which addressed the unprecedented challenges of the COVID-19 pandemic, particularly in areas including provision of supplies, patient and provider support, and development and dissemination of new vaccine and drug candidates.

**Methods:**

We examined peer reviewed and grey literature from September 2020 to June 2021 to describe the relationship between the U.S. healthcare system and Military Health System (MHS). For analysis, we applied the World Health Organization framework for health systems, which consists of six building blocks.

**Results:**

The strongest collaborative efforts occurred in areas of medicine and technology, human resources, and healthcare delivery, most notably in the MHS supplying providers, setting up treatment venues, and participating in development of vaccines and therapeutics. Highlighting that the MHS, with its centralized structure and ability to deploy assets rapidly, is an important contributor to the nation’s ability to provide a coordinated, large-scale response to health emergencies.

**Conclusions:**

Continuing the relationship between the two health systems is vital to maintaining the nation’s capability to meet future health challenges.

**Supplementary Information:**

The online version contains supplementary material available at 10.1186/s12961-022-00885-4.

## Introduction

The U.S.COVID-19 pandemic has caused more than one million deaths in the United States (U.S.) as of June 2022 [1]. It was first declared a public health emergency (PHE) on January, 31, 2020 [2]. Since then, its rapid and continued spread has posed challenges in nearly all facets of the U.S. economy, most notably in the healthcare sector, which has struggled with bed capacity, provider availability, and supply chain issues to meet the needs of patients [3]. Provision of civilian health services is one of the largest industries in the U.S., composed of a fragmented delivery system organized at the local level and funded by a mix of private and federally funded insurance programs [4, 5]. While the U.S. civilian healthcare system boasts a multitude of strengths, including its large highly trained workforce, wide range of medical specialists, secondary and tertiary institutions, cutting edge medical equipment, and for some medical services, unparalleled health outcomes, it is frequently overburdened and cost-inefficient even during normal operations [6, 7]. The additional challenges posed by the COVID-19 PHE spurred collaborative efforts by the U.S. civilian healthcare sector with the U.S. Military Health System (MHS) in the national response to the COVID-19 pandemic [8, 9]. Such collaboration has traditionally occurred through healthcare training programs, or in times of natural or other disasters, though on a much smaller scale [10–13].

The MHS is a complex system that weaves together healthcare delivery, medical education, public health, private sector partnerships, and cutting edge medical research and development with personnel and infrastructure from the Military Departments (Army, Navy, and Air Force) and the office of the Assistant Secretary of Defense (e.g., the Defense Health Agency [DHA], and the Uniformed Services University of the Health Sciences.)[14] The MHS serves approximately 9.6 million beneficiaries, of whom approximately 17% are active-duty service members from every military branch, with the remainder being non-active-duty dependents and retirees, representative of the U.S. population [15]. The MHS is focused on deployment readiness with the ability to mobilize assets and resources efficiently in support of national public health emergencies. This paper explores the inter-relationship between the U.S. healthcare system and the MHS through the lens of a health systems approach. The World Health Organization’s (WHO) Health Systems Framework breaks down a health system into six building blocks: (1) governance, (2) medicine and technology, (3) human resources, (4) healthcare delivery, (5) finances, and (6) information [16]. A health systems approach enables a comprehensive and detailed review organized according to these building blocks, and illustrates lessons learned from the MHS support to the U.S. civilian health system during a time of global pandemic.

## Methods

A review of the peer-reviewed and grey literature was conducted to examine the inter-relationship between the U.S. non-federal healthcare services and the MHS throughout the COVID-19 PHE. The six building blocks of a health system, as defined by the WHO, and shown in Table 1 below, were used to frame our findings and discussion [16].

**Table 1:** Description of WHO Building Blocks Used in this Analysis

Governance	Strategic policy frameworks combined with effective oversight, coalition-building, regulation, attention to system design and accountability
Medicine and Technology	Equitable access to essential medical products, vaccines and technologies or assured quality, safety, efficacy and cost-effectiveness, and their scientifically sound and cost-effective use
Human Resources	All personnel involved in the process of providing care and running healthcare facilities
Healthcare Delivery	Coverage, access, quality, and safety of health services
Healthcare Financing	Funds necessary to ensure people have access to needed health services
Healthcare Information	Technology used to store, transmit, and use healthcare information

Resources were identified through scanning the electronic search engines (Google, Twitter, PubMed) between September 2020 and June 2021 using terms specific to each health systems building block for the U.S. and MHS (see Additional file 1). Further, snowball sampling was employed during the literature review process within each building block to identify additional resources for review and inclusion. Studies were organized and described within the building blocks of health systems with an eye at drawing conclusions around the inter-relationship of the MHS and U.S. health systems.

## Results

### Governance

In comparison to the centralized governance approach guided by the National Security Strategy, the U.S. health services system is highly fragmented with many organizations involved in its governance, including Congress, the Department of Health & Human Services (DHHS), state and local public health departments, and the private sector healthcare insurers and systems [4, 5]. Governance of each body sculpted their response to the COVID-19 PHE, with the U.S. civilian healthcare system response largely shaped at the state or local level for initial decisions on lock down/stay at home directives, access to testing, and mask-wearing guidelines and messaging [17, 18]. In contrast, the more centralized nature of the MHS led to more consistent messaging, including the rollout of standardized language and guidelines around Force Health Protection Conditions, using the provider-based Clinical Communities Advisory Council as a bi-weekly sounding board [19]. Additionally, the development of MHS clinical practice guidelines for the treatment of COVID-19 was built on the existing platforms for the Joint Trauma System, enabling rapid development and dissemination of guidelines on a system-wide level [20].

The MHS provided coordinated support on request to state and local-level civilian authorities through the joint doctrine Defense Support To Civil Authorities (DSCA) [21]. As a result, the MHS retained its centralized communications but generally followed state-level guidance.

### Medicine and technology

Medicine and technology have been vital aspects of the nation’s response to the COVID-19 public health emergency, most notably in Operation Warp Speed (OWS), a $10 billion initiative which aimed to forge “a partnership between DHHS, the Department of Defense (DoD), and the private sector focused on developing, producing, and distributing materials for testing, prevention, and therapy on a rapid timeline.”[8] Specifically, the role of the DoD as a co-lead was to provide leadership through logistical expertise, including program management and contracting proficiency [22]. The DoD also contributed to research efforts alongside manufacturers from the U.S. healthcare sector, including clinical trial testing at MHS operated military treatment facilities [23]. Notably, the U.S. Army Research Institute for Infectious Diseases, in conjunction with pharmaceutical manufacturer Gilead, performed necessary testing for Gilead’s existing drug Remdesivir to receive Food and Drug Administration approval as a potential treatment for COVID-19 [24, 25]. As of June 2021, three vaccines had been awarded emergency use authorizations, and were distributed to adults and children over 12 across the U.S [26]. As of June 2021, OWS contributed to the distribution of over 372 million vaccines nationwide, including 4 million vaccines to DoD personnel [27, 28]. Aside from OWS, the military has also engaged in research of its own showing how medical practices in the military can inform civilian public health practices, a specific example being the use of military experience with different testing strategies and ways to mitigate the spread of the virus in close quarters and applying such experience to civilian college dormitories, prisons, and sports training environments [29].

### Human resources

The U.S. has a highly skilled workforce, but staff shortages exist and are expected to grow in the coming years [30, 31]. The COVID-19 pandemic exacerbated this staffing shortage due to increased volume of patients and illness among providers [32, 33]. Critical staffing shortages drove state and local civilian authorities to seek federal assistance through DoD provider support to civilian hospitals. Approximately 160 Air Force medical and support personnel deployed to California, and 580 Army and Navy medical and support personnel deployed to Texas [34]. An additional team of 85 personnel from the U.S. Army Urban Augmentation Task Force, 44-person acute care team and four, seven-person Rapid Rural Response Teams from the Navy were activated to support efforts in Texas [34]. Additionally, two U.S. Naval Ships, the U.S.N.S. Comfort and U.S.N.S. Mercy, deployed to New York and Los Angeles respectively providing healthcare personnel [35]. The mission was originally to provide care to non-COVID-19 patients, however, the U.S.N.S. Comfort soon took on infected patients. Governors across numerous states mobilized National Guard members to bolster vaccination efforts [36]. Overall, over 20,000 National Guardsmen deployed in 52 states and territories and other military personnel are prepared to deploy and respond within 48 h’ notice to any requests from DHHS [37].

The MHS also faced staffing challenges of its own, with the Army reaching out to retired military doctors and medics to return to service and provide care in Military Treatment Facilities (MTFs), while troops were deployed to field settings to combat the spread of COVID-19 both in the U.S. and abroad [38–40]. Additionally, the MHS determined that there were not enough trained personnel to fill key pandemic response leadership roles and that more training was warranted to produce these individuals [41]. The cooperation between U.S. civilian healthcare workers and military healthcare workers has proven crucial in ensuring adequate healthcare provider staffing levels throughout the pandemic [42]. In October 2020, the death toll from COVID-19 among nurses equaled that among nurses in World War I even with data available from only a quarter of countries and the numbers have only increased since [43].

### Healthcare delivery

Intricately linked to its role in human resources, the MHS has provided support to the U.S. civilian healthcare system through enabling additional delivery of services. Outpatient appointments, inpatient admissions, and surgeries dramatically decreased among patients with commercial insurance, Medicare, Medicaid, and the MHS-provided TRICARE [44–46]. While this freed some bed capacity to care for COVID-19 patients, the U.S. has fewer physicians and hospital beds per capita compared to other countries already overwhelmed by COVID-19, and a higher population of patients who required intensive care [33, 47]. The MHS was able to aid the civilian healthcare system by setting up field hospitals to care for civilian patients [48].

This support was provided in part through joint doctrine of DSCA as described in Governance, above [21]. Three major examples were the standup of the field hospital at the Jacob J. Javitz Convention Center in New York City in June 2020, which enabled treatment of approximately 1100 patients, the deployment of the two Naval Ships, U.S.N.S. Comfort and U.S.N.S. Mercy, to New York and Los Angeles respectively in May 2020 and the admission of non-COVID-19 patients outside the MHS to William Beaumont Army Medical Center in order to free beds in other El Paso hospital facilities in October 2020 [35, 49–51].

The MHS partnered with civilian entities through OWS, providing expertise in the areas of operational planning, logistics and supply chains [22, 52]. The MHS leveraged its contracting connections to obtain deals with the six manufacturers, three of which have developed vaccines currently in use [22]. The MHS also supported vaccination distribution logistics and provided manpower [22, 52]. These examples show how the rapid deployment of MHS personnel and military assets provided vital support to the U.S. civilian healthcare system during the pandemic.

### Healthcare financing

The U.S. civilian healthcare system consumes 17.8% of the GDP and is the most expensive per capita in the world [53]. The MHS normally costs approximately $52 billion per year and has been holding steady at 10% of the DoD total budget since 2010 [54]. The COVID-19 pandemic took a large toll on the financing of the U.S. civilian healthcare system. While demand for specialty care to treat COVID-19 increased, demand for routine services decreased dramatically leading to reduced revenues for providers and hospitals. U.S. civilian hospitals were estimated to lose $323.1 billion in 2020 [55]. Additionally, many individuals who were laid off due to the pandemic lost their health insurance coverage and were left unable to pay to receive medical care [55]. The MHS also reduced healthcare expenditures during the pandemic due to the reduction in direct and purchased care utilization, but for the MHS this is positive savings [56]. However, there were still issues centered around financial lines of communication within the DHA and service resources. Issues occurred when it was not clear which party was responsible for funding, creating spending plans, submitting and coordinating funding requests, determining the appropriate funding source, or creating expense reports. In some cases, these financial issues caused mission delays [56]. No collaborative efforts were found during this time period between the MHS and the U.S. civilian healthcare system regarding financing.

### Healthcare information

Healthcare information technology in the U.S. is composed of hundreds of electronic health record (EHR) systems, data repositories, and information systems. There is a need for increased health information exchanges across the country to improve quality of care and the COVID-19 pandemic encouraged more data sharing than previously existed.

The Tiberius platform developed by Palantir Technologies specifically for OWS response to the COVID-19 pandemic, is used to collect, correlate, and visualize data across OWS for military and civilian partners spanning the MHS as well as civilian public health and medical networks. This platform integrates data on manufacturing, supply chain, allocation, state and territory planning, and administration of the vaccine, however, no identifiable data or personal health information is contained in the platform [41]. Another useful platform, GeoHealth, a geographic information system application sponsored by the Office of the Assistant Secretary of Defense for Preparedness and Response, is used by military and civilian entities to examine burden of disease, population-level risk factors, and bed and ventilator capacity of hospitals at the county level across the U.S [57]. Both platforms have proven invaluable in tracking capacity for treatment and prevention of COVID-19.

Sharing of EHR data has been critically important when treating complex COVID-19 patients however, the majority of EHR systems are not interconnected or interoperable. To overcome this, the Joint Health Information Exchange, which is a military based data exchange, expanded to allow bidirectional data sharing from CommonWell Health Alliance, a civilian entity spanning over 15,000 hospitals and clinics [58, 59]. This data sharing allows for more efficient treatment of COVID-19 patients.

## Discussion

The MHS, serves a representative population of Americans and is a crucial player in the multipronged approach to filling in the gaps in the U.S. civilian healthcare sector that have been exposed during both the PHE and pandemic as a whole. The timing and variation in the spread of COVID-19 across the U.S. has required substantial agility in the mobilization of resources, which the military medical forces were equipped to provide. This capability is supported by a well-developed research capacity, which contributed significantly to the development and testing of vaccines and therapeutics, and a dedicated logistics infrastructure which enabled rapid delivery of vaccines during OWS [22, 52]. Overall, the MHS played a vital role in the U.S. civilian healthcare sector response to the COVID-19 pandemic, with the most pronounced collaborative efforts seen in human resources, healthcare delivery, and medicine and technology.

The areas of governance, finance, and information technology presented fewer opportunities for collaborative efforts, due to fundamental differences in priorities and administration between the two systems. For instance, the varied state-level responses to the pandemic highlighted the fragmentation of care inherent throughout the U.S. civilian healthcare sector, contrasting with the centralized, mission-focused response of the MHS. Regarding healthcare financing, no collaborative efforts were found during this time period between the MHS and the U.S. civilian healthcare system, though this could change if these efforts continue. Communication between each system’s disparate information systems remains a significant challenge, and future data sharing should be addressed to ease future efforts.

Within the areas of human resources, healthcare delivery, and medicine and technology, the collaborative efforts between the MHS and U.S. civilian healthcare system provides important lessons for the continued response to the COVID-19 pandemic and future national health emergencies, including the need to continue training new generations of providers to ensure enough are available to respond, retaining capabilities for mobile medical treatment and testing facilities in the case that permanent structures are full, and continued scientific collaborative efforts between the MHS and U.S. civilian healthcare system. Together, both systems have a wealth of knowledge, skill, and ability that can work in tandem to better care for our nation, develop therapeutics, and administer them efficiently.

### Limitations

This study had several limitations. First, the evolving nature of the COVID-19 pandemic means that knowledge and new literature are generated daily, and collaborative efforts between the U.S. healthcare system and the MHS continue to change in response to emerging priorities. Second, while this study presents lessons learned, it does not address hard outcomes in terms of morbidity and mortality, health service utilization, or costs. Finally, this study does not include late-breaking changes driven by the wide release of COVID-19 vaccines. Thus, this paper is intended to serve as a snapshot in time of the pandemic prior to June 2021.

## Conclusion

The COVID-19 PHE has demonstrated that the U.S. civilian healthcare sector depends on the MHS and its assets to rapidly fill gaps and strengthen its capacity. The COVID-19 PHE demonstrated that the U.S. depended on the MHS and its assets in its response. It needed MHS personnel to provide direct support to hot spots across the country, to provide leadership alongside DHHS in OWS, and support regulatory social distancing. Further, our findings highlight that MHS providers have been essential in supporting their civilian counterparts during the COVID-19 PHE. Overall, the COVID-19 PHE highlighted the MHS as instrumental to the collaboration needed to address the pandemic. Therefore continuing to further develop and strengthen this partnership is necessary in preparation for continued, timely response to future PHEs.

## Supplementary Information

**Additional file 1:** Complete list of search terms used during data collection.

## Data Availability

All information pertaining to our research is contained within the manuscript; no external datasets were used.

## Abbreviations

DHHS: Department of Health & Human Services
DoD: U.S. Department of Defense
DSCA: Defense Support To Civil Authorities
EHR: Electronic health record
GDP: Gross domestic product
MHS: Military Health System
MTF: Military treatment facility
OWS: Operation warp speed
PHE: Public Health Emergency
WHO: World Health Organization
U.S.: United States of America
U.S.N.S.: U.S. Naval Ship
