# Supplementary material for: The collaborative research and service delivery partnership between the United States healthcare system and the U.S. Military Health System during the COVID-19 pandemic
Source: Health Res Policy Syst. 2022 Jul 19;20:81. doi: 10.1186/s12961-022-00885-4 (PMC9295105; doi:10.1186/s12961-022-00885-4)
Supplement: Supplementary file 1 — Additional file 1: Complete list of search terms used during data collection. [file 12961_2022_885_MOESM1_ESM.docx]

**Additional File 1.**

**List of Search Terms**

MHS Governance

MHS Governance COVID

MHS Governance Coronavirus

Military Health System Governance

Military Health System COVID

Military Health System Coronavirus

MHS Policy

MHS Policy COVID

MHS Policy Coronavirus

Military Health System Policy

Military Health System Policy COVID

Military Health System Policy Coronavirus

MHS Regulation

MHS Regulation COVID

MHS Regulation Coronavirus

Military Health System Regulation

Military Health System Regulation COVID

Military Health System Regulation Coronavirus

MHS Oversight

MHS Oversight COVID

MHS Oversight Coronavirus

Military Health System Oversight

Military Health System Oversight COVID

Military Health System Oversight Coronavirus

US Health System Governance

US Health System Governance COVID

US Health System Governance Coronavirus

United States Health System Governance

United States Health System Governance COVID

United States Health System Governance Coronavirus

US Health Care Governance

US Health Care Governance COVID

US Health Care Governance Coronavirus

United States Health Care Governance

United States Health Care Governance COVID

United States Health Care Governance Coronavirus

US Health System Policy

US Health System Policy COVID

US Health System Policy Coronavirus

United States Health System Policy

United States Health System Policy COVID

United States Health System Policy Coronavirus

US Health Care Policy

US Health Care Policy COVID

US Health Care Policy Coronavirus

United States Health Care Policy

United States Health Care Policy COVID

United States Health Care Policy Coronavirus

US Health System Regulation

US Health System Regulation COVID

US Health System Regulation Coronavirus

United States Health System Regulation

United States Health System Regulation COVID

United States Health System Regulation Coronavirus

US Health Care Regulation

US Health Care Regulation COVID

US Health Care Regulation Coronavirus

United States Health Care Regulation

United States Health Care Regulation COVID

United States Health Care Regulation Coronavirus

US Health System Oversight

US Health System Oversight COVID

US Health System Oversight Coronavirus

United States Health System Oversight

United States Health System Oversight COVID

United States Health System Oversight Coronavirus

US Health Care Oversight

US Health Care Oversight COVID

US Health Care Oversight Coronavirus

United States Health Care Oversight

United States Health Care Oversight COVID

United States Health Care Oversight Coronavirus

MHS Medicine

MHS Medicine COVID

MHS Medicine Coronavirus

Military Health System Medicine

Military Health System Medicine COVID

Military Health System Medicine Coronavirus

MHS Technology

MHS Technology COVID

MHS Technology Coronavirus

Military Health System Technology

Military Health System Technology COVID

Military Health System Technology Coronavirus

MHS Vaccines

MHS Vaccines COVID

MHS Vaccines Coronavirus

Military Health System Vaccines

Military Health System Vaccines COVID

Military Health System Vaccines Coronavirus

MHS Treatments

MHS Treatments COVID

MHS Treatments Coronavirus

Military Health System Treatments

Military Health System Treatments COVID

Military Health System Treatments Coronavirus

MHS Access

MHS Access COVID

MHS Access Coronavirus

Military Health System Access

Military Health System Access COVID

Military Health System Access Coronavirus

US Health System Medicine

US Health System Medicine COVID

US Health System Medicine Coronavirus

United States Health System Medicine

United States Health System Medicine COVID

United States Health System Medicine Coronavirus

US Health Care Medicine

US Health Care Medicine COVID

US Health Care Medicine Coronavirus

United States Health Care Medicine

United States Health Care Medicine COVID

United States Health Care Medicine Coronavirus

US Health System Technology

US Health System Technology COVID

US Health System Technology Coronavirus

United States Health System Technology

United States Health System Technology COVID

United States Health System Technology Coronavirus

US Health Care Technology

US Health Care Technology COVID

US Health Care Technology Coronavirus

United States Health Care Technology

United States Health Care Technology COVID

United States Health Care Technology Coronavirus

US Health System Vaccines

US Health System Vaccines COVID

US Health System Vaccines Coronavirus

United States Health System Vaccines

United States Health System Vaccines COVID

United States Health System Vaccines Coronavirus

US Health Care Vaccines

US Health Care Vaccines COVID

US Health Care Vaccines Coronavirus

United States Health Care Vaccines

United States Health Care Vaccines COVID

United States Health Care Vaccines Coronavirus

US Health System Treatments

US Health System Treatments COVID

US Health System Treatments Coronavirus

United States Health System Treatments

United States Health System Treatments COVID

United States Health System Treatments Coronavirus

US Health Care Treatments

US Health Care Treatments COVID

US Health Care Treatments Coronavirus

United States Health Care Treatments

United States Health Care Treatments COVID

United States Health Care Treatments Coronavirus

US Health System Access

US Health System Access COVID

US Health System Access Coronavirus

United States Health System Access

United States Health System Access COVID

United States Health System Access Coronavirus

US Health Care Access

US Health Care Access COVID

US Health Care Access Coronavirus

United States Health Care Access

United States Health Care Access COVID

United States Health Care Access Coronavirus

MHS Human Resources

MHS Human Resources COVID

MHS Human Resources Coronavirus

Military Health System Human Resources

Military Health System Human Resources COVID

Military Health System Human Resources Coronavirus

MHS Personnel

MHS Personnel COVID

MHS Personnel Coronavirus

Military Health System Personnel

Military Health System Personnel COVID

Military Health System Personnel Coronavirus

MHS Providers

MHS Providers COVID

MHS Providers Coronavirus

Military Health System Providers

Military Health System Providers COVID

Military Health System Providers Coronavirus

MHS Nurses

MHS Nurses COVID

MHS Nurses Coronavirus

Military Health System Nurses

Military Health System Nurses COVID

Military Health System Nurses Coronavirus

MHS Facilities

MHS Facilities COVID

MHS Facilities Coronavirus

Military Health System Facilities

Military Health System Facilities COVID

Military Health System Facilities Coronavirus

US Health System Human Resources

US Health System Human Resources COVID

US Health System Human Resources Coronavirus

United States Health System Human Resources

United States Health System Human Resources COVID

United States Health System Human Resources Coronavirus

US Health Care Human Resources

US Health Care Human Resources COVID

US Health Care Human Resources Coronavirus

United States Health Care Human Resources

United States Health Care Human Resources COVID

United States Health Care Human Resources Coronavirus

US Health System Personnel

US Health System Personnel COVID

US Health System Personnel Coronavirus

United States Health System Personnel

United States Health System Personnel COVID

United States Health System Personnel Coronavirus

US Health Care Personnel

US Health Care Personnel COVID

US Health Care Personnel Coronavirus

United States Health Care Personnel

United States Health Care Personnel COVID

United States Health Care Personnel Coronavirus

US Health System Providers

US Health System Providers COVID

US Health System Providers Coronavirus

United States Health System Providers

United States Health System Providers COVID

United States Health System Providers Coronavirus

US Health Care Providers

US Health Care Providers COVID

US Health Care Providers Coronavirus

United States Health Care Providers

United States Health Care Providers COVID

United States Health Care Providers Coronavirus

US Health System Nurses

US Health System Nurses COVID

US Health System Nurses Coronavirus

United States Health System Nurses

United States Health System Nurses COVID

United States Health System Nurses Coronavirus

US Health System Facilities

US Health System Facilities COVID

US Health System Facilities Coronavirus

United States Health System Facilities

United States Health System Facilities COVID

United States Health System Facilities Coronavirus

US Health Care Facilities

US Health Care Facilities COVID

US Health Care Facilities Coronavirus

United States Health Care Facilities

United States Health Care Facilities COVID

United States Health Care Facilities Coronavirus

MHS Coverage

MHS Coverage COVID

MHS Coverage Coronavirus

Military Health System Coverage

Military Health System Coverage COVID

Military Health System Coverage Coronavirus

MHS Quality Care

MHS Quality Care COVID

MHS Quality Care Coronavirus

Military Health System Quality Care

Military Health System Quality Care COVID

Military Health System Quality Care Coronavirus

MHS Safety

MHS Safety COVID

MHS Safety Coronavirus

Military Health System Safety

Military Health System Safety COVID

Military Health System Safety Coronavirus

MHS Health Services

MHS Health Services COVID

MHS Health Services Coronavirus

Military Health System Health Services

Military Health System Health Services COVID

Military Health System Health Services Coronavirus

US Health System Coverage

US Health System Coverage COVID

US Health System Coverage Coronavirus

United States Health System Coverage

United States Health System Coverage COVID

United States Health System Coverage Coronavirus

US Health Care Coverage

US Health Care Coverage COVID

US Health Care Coverage Coronavirus

United States Health Care Coverage

United States Health Care Coverage COVID

United States Health Care Coverage Coronavirus

US Health System Quality Care

US Health System Quality Care COVID

US Health System Quality Care Coronavirus

United States Health System Quality Care

United States Health System Quality Care COVID

United States Health System Quality Care Coronavirus

US Health Care Quality Care

US Health Care Quality Care COVID

US Health Care Quality Care Coronavirus

United States Health Care Quality Care

United States Health Care Quality Care COVID

United States Health Care Quality Care Coronavirus

US Health System Safety

US Health System Safety COVID

US Health System Safety Coronavirus

United States Health System Safety

United States Health System Safety COVID

United States Health System Safety Coronavirus

US Health Care Safety

US Health Care Safety COVID

US Health Care Safety Coronavirus

United States Health Care Safety

United States Health Care Safety COVID

United States Health Care Safety Coronavirus

US Health System Health Services

US Health System Health Services COVID

US Health System Health Services Coronavirus

United States Health System Health Services

United States Health System Health Services COVID

United States Health System Health Services Coronavirus

US Health Care Health Services

US Health Care Health Services COVID

US Health Care Health Services Coronavirus

United States Health Care Health Services

United States Health Care Health Services COVID

United States Health Care Health Services Coronavirus

MHS Financing

MHS Financing COVID

MHS Financing Coronavirus

Military Health System Financing

Military Health System Financing COVID

Military Health System Financing Coronavirus

MHS Finances

MHS Finances COVID

MHS Finances Coronavirus

Military Health System Finances

Military Health System Finances COVID

Military Health System Finances Coronavirus

MHS Costs

MHS Costs COVID

MHS Costs Coronavirus

Military Health System Costs

Military Health System Costs COVID

Military Health System Costs Coronavirus

MHS Funding

MHS Funding COVID

MHS Funding Coronavirus

Military Health System Funding

Military Health System Funding COVID

Military Health System Funding Coronavirus

US Health System Financing

US Health System Financing COVID

US Health System Financing Coronavirus

United States Health System Financing

United States Health System Financing COVID

United States Health System Financing Coronavirus

US Health Care Financing

US Health Care Financing COVID

US Health Care Financing Coronavirus

United States Health Care Financing

United States Health Care Financing COVID

United States Health Care Financing Coronavirus

US Health System Finances

US Health System Finances COVID

US Health System Finances Coronavirus

United States Health System Finances

United States Health System Finances COVID

United States Health System Finances Coronavirus

US Health Care Finances

US Health Care Finances COVID

US Health Care Finances Coronavirus

United States Health Care Finances

United States Health Care Finances COVID

United States Health Care Finances Coronavirus

US Health System Costs

US Health System Costs COVID

US Health System Costs Coronavirus

United States Health System Costs

United States Health System Costs COVID

United States Health System Costs Coronavirus

US Health Care Costs

US Health Care Costs COVID

US Health Care Costs Coronavirus

United States Health Care Costs

United States Health Care Costs COVID

United States Health Care Costs Coronavirus

US Health System Funding

US Health System Funding COVID

US Health System Funding Coronavirus

United States Health System Funding

United States Health System Funding COVID

United States Health System Funding Coronavirus

US Health Care Funding

US Health Care Funding COVID

US Health Care Funding Coronavirus

United States Health Care Funding

United States Health Care Funding COVID

United States Health Care Funding Coronavirus

MHS Health Care Information

MHS Health Care Information COVID

MHS Health Care Information Coronavirus

Military Health System Health Care Information

Military Health System Health Care Information COVID

Military Health System Health Care Information Coronavirus

MHS Electronic Health Record

MHS Electronic Health Record COVID

MHS Electronic Health Record Coronavirus

Military Health System Health Record

Military Health System Health Record COVID

Military Health System Health Record Coronavirus

MHS Health Records

MHS Health Records COVID

MHS Health Records Coronavirus

Military Health System Health Records

Military Health System Health Records COVID

Military Health System Health Records Coronavirus

US Health System Health Care Information

US Health System Health Care Information COVID

US Health System Health Care Information Coronavirus

United States Health System Health Care Information

United States Health System Health Care Information COVID

United States Health System Health Care Information Coronavirus

US Health Care Health Care Information

US Health Care Health Care Information COVID

US Health Care Health Care Information Coronavirus

United States Health Care Health Care Information

United States Health Care Health Care Information COVID

United States Health Care Health Care Information Coronavirus

US Health System Electronic Health Record

US Health System Electronic Health Record COVID

US Health System Electronic Health Record Coronavirus

United States Health System Electronic Health Record

United States Health System Electronic Health Record COVID

United States Health System Electronic Health Record Coronavirus

US Health Care Electronic Health Record

US Health Care Electronic Health Record COVID

US Health Care Electronic Health Record Coronavirus

United States Health Care Electronic Health Record

United States Health Care Electronic Health Record COVID

United States Health Care Electronic Health Record Coronavirus

US Health System Health Records

US Health System Health Records COVID

US Health System Health Records Coronavirus

United States Health System Health Records

United States Health System Health Records COVID

United States Health System Health Records Coronavirus

US Health Care Health Records

US Health Care Health Records COVID

US Health Care Health Records Coronavirus

United States Health Care Health Records

United States Health Care Health Records COVID

United States Health Care Health Records Coronavirus
